# Acute Renal Infarction Secondary to Superior Polar Artery Thrombosis: A Case Report

**DOI:** 10.7759/cureus.105474

**Published:** 2026-03-18

**Authors:** Ramesh Karki, Kolhe Lokesh Krishnaji, Nanthini Kunaratnam

**Affiliations:** 1 Emergency Medicine, Woodlands Hospital, Singapore, SGP

**Keywords:** anatomical variant, anticoagulation, polar artery, renal infarction, thrombosis

## Abstract

Acute renal infarction is a rare condition, often misdiagnosed due to a nonspecific presentation. Anatomical variants of renal arteries may predispose to atypical infarction patterns and influence clinical outcomes. We report a 55-year-old Thai male patient who presented with sudden-onset severe left iliac fossa pain. Initial evaluation revealed leukocytosis, proteinuria, microscopic hematuria, and elevated lactate. Contrast-enhanced computed tomography demonstrated acute thrombosis of a variant superior polar artery with resultant wedge-shaped infarction of the upper renal pole. The patient had early bifurcation of the left main renal artery into dominant superior polar and inferior arteries. Treatment consisted of systemic anticoagulation with unfractionated heparin followed by enoxaparin. The patient recovered without complications, maintaining normal renal function at follow-up. This case highlights the importance of recognising renal infarction in patients with acute abdominal pain, particularly when anatomical variants are present. Early diagnosis through appropriate imaging and prompt anticoagulation can preserve renal function and prevent complications. Clinicians should maintain a high suspicion for renal infarction in patients with sudden severe pain and suggestive laboratory findings.

## Introduction

Acute renal infarction represents a vascular emergency with significant potential for permanent renal damage if diagnosis is delayed. This condition is frequently overlooked due to its nonspecific clinical presentation that mimics more common abdominal pathologies such as renal colic, appendicitis, or bowel obstruction [1-3]. 

The majority of renal infarctions result from thromboembolic events, with atrial fibrillation being the most common predisposing factor, present in up to 91% of cases [1,4-6]. However, anatomical variants of the renal arterial system may create unique predispositions to thrombosis and alter the typical presentation patterns. Approximately 25-30% of individuals possess accessory or variant renal arteries, including polar arteries that supply specific renal segments.

Early recognition of renal infarction is crucial, as prompt anticoagulation can limit infarct size and preserve renal function. Laboratory markers such as elevated lactate dehydrogenase (LDH) present in 90-94% of cases, combined with hematuria and leukocytosis, should raise clinical suspicion [1,2,4,7-13]. Contrast-enhanced computed tomography (CT) is the preferred imaging modality for the diagnosis of renal infarction, with high diagnostic sensitivity and specificity and reported diagnostic accuracy approaching 100% [1,11].

We present a case of acute renal infarction secondary to thrombosis of a variant superior polar artery, emphasising the diagnostic challenges and management considerations in patients with anatomical variants.

## Case presentation

A 55-year-old Thai male construction worker presented to our emergency department with sudden-onset severe left-sided abdominal pain. He had no significant past medical history, took no regular medications, and denied smoking or recreational drug use. Family history was unremarkable for cardiovascular or thromboembolic disorders.

The patient described the pain as sudden in onset, localised to the left iliac fossa with a sharp, constant quality rated 9/10 in severity. The pain did not radiate and was not relieved by position changes. He experienced two episodes of non-bilious vomiting but denied urinary symptoms, fever or similar previous episodes.

On examination, the patient appeared distressed and mildly dehydrated. Vital signs revealed blood pressure 145/92 mmHg, heart rate 98 beats per minute (regular), temperature 37.2°C, respiratory rate 18 breaths per minute, and oxygen saturation 98% on room air. Abdominal examination demonstrated marked tenderness with voluntary guarding and rebound tenderness in the left iliac fossa. Bowel sounds were present but diminished. Costovertebral angle tenderness was absent bilaterally. Cardiovascular examination revealed regular rhythm without murmurs, and peripheral pulses were palpable and symmetrical with no audible bruits. Respiratory, neurological, and genitourinary examinations were unremarkable.

Initial laboratory investigations revealed leukocytosis with a white blood cell count of 14.1 × 10⁹/L (reference range, 4.0-11.0), haemoglobin 13.5 g/dL, and platelet count 285 × 10⁹/L. Renal function showed mild impairment with urea 5.5 mmol/L (reference range, 2.5-7.5) and creatinine 106 μmol/L (reference range, 60-110). Electrolytes were within normal limits; potassium 4 mmol/L. Urinalysis demonstrated 2+ hematuria and 3+ proteinuria without leukocytes or nitrites. Venous blood gas analysis showed pH 7.45 with elevated LDH of 2.63 mmol/L (reference range, <2.0). Coagulation studies were normal with an international normalised ratio (INR) of 0.9 and activated partial thromboplastin time (aPTT) of 24.6 seconds. ECG showed a 70/minute sinus rhythm with no ischemic changes. Point-of-care ultrasound excluded hydro nephrosis and abdominal aortic aneurysm, though gallbladder sludge was noted. Given the clinical presentation and laboratory findings suggestive of possible renal pathology, urgent contrast-enhanced CT of the abdomen and pelvis was performed.

**Figure 1:**
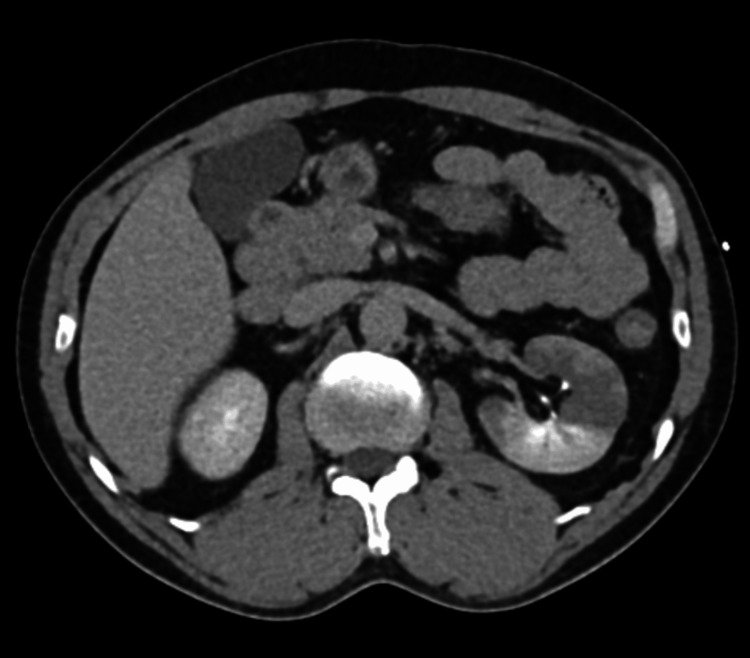
CT (axial view) showing acute infarction of the superior pole of the kidney with wedge-shaped perfusion defect secondary to thrombosis of variant superior polar artery

**Figure 2:**
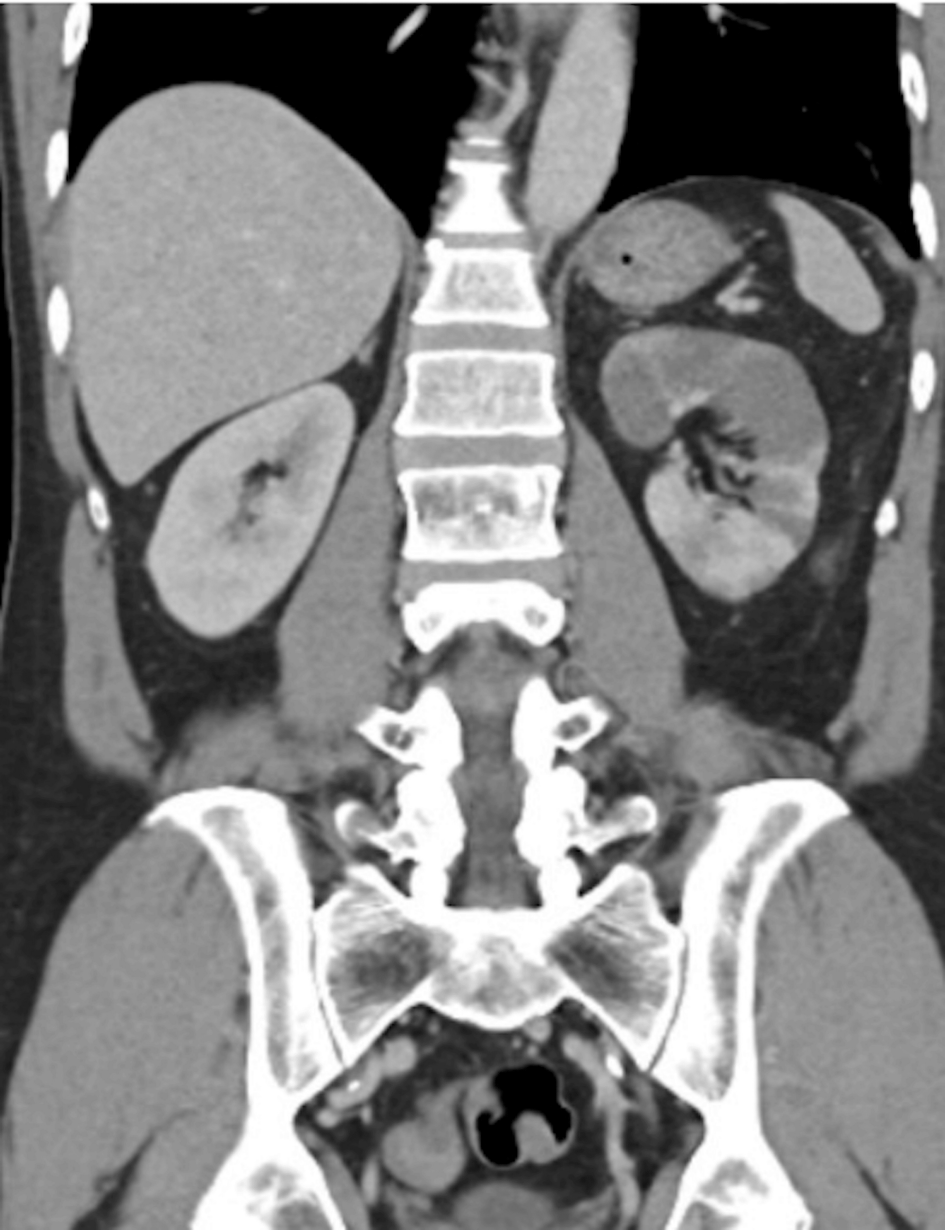
CT (coronal view) showing acute infarction of the superior pole of the kidney with wedge-shaped perfusion defect secondary to thrombosis of variant superior polar artery

CT imaging revealed an acute infarction of the superior pole of the left kidney secondary to thrombosis of a variant superior polar artery (Figures 1, 2). The left main renal artery demonstrated early bifurcation into a dominant superior polar artery and an inferior renal artery. There was an abrupt occlusion of the superior polar artery with associated wedge-shaped perfusion defect involving approximately 40% of the renal parenchyma and mild swelling of the affected segment. The inferior renal artery and remaining kidney parenchyma showed normal enhancement. No evidence of renal artery dissection or other vascular abnormalities was identified.

Following CT confirmation of renal infarction, the vascular surgery team was urgently consulted. Given the extent of infarction and time from symptom onset (approximately eight hours), Endovascular intervention was deemed unlikely to provide benefit, and the risk of reperfusion injury was considered significant. The patient was commenced on therapeutic anticoagulation with intravenous unfractionated heparin (80 units/kg bolus followed by 18 units/kg/hour infusion) with monitoring of activated partial thromboplastin time. Supportive care included intravenous fluid resuscitation, pain management with opioid analgesia, and close monitoring of renal function and hemodynamic status. The patient was admitted to the high-dependency unit for continuous monitoring.

Further investigations to identify underlying thrombophilic conditions or embolic sources were undertaken. Transthoracic echocardiography with bubble study demonstrated normal left ventricular function (ejection fraction 65%) with mild mitral and tricuspid regurgitation. No intracardiac thrombus or patent foramen ovale was identified. Lower limb Doppler ultrasound revealed a small, partial thrombosis in the left proximal peroneal vein without evidence of deep vein thrombosis. Thrombophilia screening, including protein C, protein S, antithrombin III, factor V Leiden, and antiphospholipid antibodies, was planned for outpatient follow-up.

During the five-day hospital admission, the patient remained hemodynamically stable with gradual improvement in pain. Renal function remained stable with creatinine levels between 95-105 μmol/L. On discharge, creatinine level was 97 μmol/L Anticoagulation was transitioned to subcutaneous enoxaparin 1.5 mg/kg daily prior to discharge. Newly diagnosed hypertension (average readings 150/95 mmHg) was managed with amlodipine 5 mg daily. Long-term anticoagulation with apixaban was continued, given the thrombotic event in the absence of identified reversible risk factors.

Patient's perspective

The patient expressed initial anxiety regarding the severity of his symptoms but appreciated the thorough diagnostic approach and clear communication regarding his condition and treatment plan. He was satisfied with the rapid resolution of symptoms and return to normal activities.

## Discussion

This case illustrates several important aspects of renal infarction, particularly in the context of anatomical variants. The patient's presentation with sudden severe abdominal pain, leukocytosis, hematuria, and elevated lactate created a clinical picture suggestive of renal infarction, though the initial localisation to the iliac fossa rather than the typical flank pain pattern [10] initially suggested alternative diagnoses.

The anatomical variant identified in this case, early bifurcation of the main renal artery into dominant superior polar and inferior arteries, represents a relatively uncommon configuration present in approximately 15-20% of individuals. This variant may predispose to thrombosis through several mechanisms [1,6]. The smaller calibre of polar arteries compared to the main renal artery creates areas of relatively slower flow, potentially promoting thrombus formation. Additionally, the acute angulation at the bifurcation point may create turbulent flow patterns that activate the coagulation cascade.

The diagnostic approach in this case highlights the importance of maintaining clinical suspicion for renal infarction despite atypical presentation. While the patient lacked the classic triad of flank pain, hematuria, and hypertension, the combination of severe abdominal pain, leukocytosis, and urinalysis abnormalities warranted advanced imaging [4,9,7,11]. The elevated lactate, while nonspecific, may reflect tissue ischaemia and should prompt consideration of vascular causes of abdominal pain [1,2,4,6-8]. Elevated LDH is a consistent finding in acute renal infarction, reported in approximately 90-94% of patients in previous studies [1,2,4,6-8,11], and may serve as an early diagnostic clue in a condition that is frequently overlooked [1,2].

The presence of hematuria (71-72% of cases) and leukocytosis (85% of cases) supported the diagnosis, while the mild elevation in creatinine (present in 53% of cases) reflected the partial nature of the infarction [1,11 ]. The management approach emphasising anticoagulation over intervention reflects current understanding that endovascular therapy is most beneficial when performed within six hours of symptom onset [5]. Beyond this window, the risks of reperfusion injury may outweigh potential benefits, particularly when a significant portion of viable parenchyma remains perfused [12,14]. The decision for conservative management was appropriate given the eight-hour delay from symptom onset and the preservation of flow through the inferior renal artery. The identification of a small peroneal vein thrombosis raises questions about underlying thrombophilic tendency, though comprehensive screening was normal. This finding may represent a coincidental discovery or suggest a systemic prothrombotic state that contributed to the renal arterial thrombosis.

Long-term outcomes in renal infarction depend largely on the extent of parenchymal loss and underlying cardiovascular risk factors. In this case, the preservation of approximately 60% of renal parenchyma through the inferior arterial system likely contributed to the maintenance of normal renal function. The development of hypertension, present in up to 40% of patients following renal infarction, requires ongoing monitoring and management. This case emphasises several key learning points for clinicians. First, renal infarction should be considered in patients with sudden severe abdominal or flank pain, particularly when accompanied by haematuria, leukocytosis, or elevated lactate [15-17]. Second, anatomical variants may predispose to atypical presentations and infarction patterns. Third, prompt imaging with contrast-enhanced CT is essential for diagnosis and treatment planning [11]. Previous case reports have emphasized that early contrast-enhanced CT is particularly important when anatomical variations in the renal arteries are present, as these variations can alter the typical location of infarction [1,6 ]. Finally, early anticoagulation remains the cornerstone of management, with interventional therapy reserved for cases presenting within the therapeutic window.

## Conclusions

Acute renal infarction secondary to polar artery thrombosis represents a rare but clinically significant condition that requires high clinical suspicion for timely diagnosis. This case demonstrates that anatomical variants may contribute to thrombotic events and may alter typical presentation patterns. The combination of appropriate clinical suspicion, targeted laboratory evaluation, and prompt imaging enables accurate diagnosis and optimal management. Early anticoagulation can preserve renal function and prevent complications, even in cases with significant anatomical variants. Clinicians should maintain awareness of this condition and its varied presentations to ensure optimal patient outcomes.
